# HSF1 promotes CD69^+^ Treg differentiation to inhibit colitis progression

**DOI:** 10.7150/thno.78078

**Published:** 2023-03-21

**Authors:** Lei Yu, Bingluo Zhou, Yiran Zhu, Ling Li, Yiming Zhong, Liyuan Zhu, Hanying Wang, Hui Chen, Jinye Xu, Tianxin Guo, Lifeng Feng, Xian Wang, Zhijian Cai, Jianli Wang, Hongchuan Jin

**Affiliations:** 1 Laboratory of Cancer Biology, Key Lab of Biotherapy in Zhejiang Province, Cancer Center of Zhejiang University, Sir Run Run Shaw hospital, School of Medicine, Zhejiang University, Hangzhou, Zhejiang, China; 2 Department of Medical Oncology, Sir Run Run Shaw hospital, School of Medicine, Zhejiang University, Hangzhou, Zhejiang, China; 3 Department of Pathology, Sir Run Run Shaw hospital, School of Medicine, Zhejiang University, Hangzhou, Zhejiang, China; 4 Department of respiratory medicine, The First People's Hospital of Xiaoshan District, Xiaoshan First Affiliated Hospital of Wenzhou Medical University, Hangzhou, 311200, Zhejiang, China; 5 Institute of Immunology, and Department of Orthopedics of the Second Affiliated Hospital, Zhejiang University School of Medicine, Hangzhou, China; 6 Institute of Immunology, and Bone Marrow Transplantation Center of the First Affiliated Hospital, Zhejiang University School of Medicine, Hangzhou, China; 7 Institute of Hematology, Zhejiang University & Zhejiang Engineering Laboratory for Stem Cell and Immunotherapy, Hangzhou, China

**Keywords:** Treg, CD69, HSF1, IBD, proteasome inhibitor

## Abstract

Regulatory T cells (Tregs) are critical for generating and maintaining peripheral tolerance. Treg-based immunotherapy is valuable for the clinical management of diseases resulting from dysregulation of immune tolerance. However, the lack of potency is a potential limitation of Treg therapy. In addition, CD69 positive-Treg (CD69^+^ Treg) represent a newly identified subset of Tregs with potent immune suppressive capability.

**Methods:** *Foxp3*^YFP-Cre^*CD69*^fl/fl^ and* CD4*^Cre^*CD69*^fl/fl^ mice were generated to determine the relevance of CD69 to Treg. Chromatin Immunoprecipitation Assay (ChIP) and luciferase Assay were performed to detect the regulation of CD69 transcription by heat shock transcription factor 1(HSF1). Gene expression was measured by western blotting and qRT-PCR. The differentiation of naive T cells to CD69^+^Foxp3^+^ iTregs was determined by flow cytometry. The immunosuppressive ability of Tregs was analyzed by ELISA and flow cytometry. Colon inflammation in mice was reflected by changes in body weight and colon length, the disease activity index (DAI), and H&E staining of colon tissues.

**Results:** Induced Tregs (iTregs) from *CD4*^Cre^*CD69*^fl/fl^ mice failed to alleviate colitis. The transcription factor HSF1 interacted with the promoter of the CD69 gene to prompt its transcription during Treg differentiation. Genetic and chemical inhibition of HSF1 impaired CD69^+^ Treg differentiation and promoted the pathogenesis of colitis in mice. In contrast, HSF1 protein stabilized by inhibiting its proteasomal degradation promoted CD69^+^ Treg differentiation and alleviated colitis in mice. Moreover, adoptive transfer of iTregs with HSF1 stabilization by proteasome inhibitor (PSI) dramatically prevented the development of colitis in mice and was accompanied by decreased production of pro-inflammatory cytokines and reduced accumulation of pro-inflammatory lymphocytes in colitis tissue, whereas Tregs induced in the absence of PSI were less stable and ineffective in suppressing colitis.

**Conclusions:** HSF1 promotes CD69^+^ Tregs differentiation by activating the CD69 transcription, which is critical for the immunosuppressive function of Tregs. Stabilization of HSF1 by PSIs results in the efficient generation of Tregs with high potency to treat colitis and probably other autoimmune diseases involving Tregs deficiency.

## Introduction

As a chronic recurrent inflammatory disease of the gastrointestinal tract, Inflammatory bowel disease (IBD) mainly including Crohn's disease (CD) and ulcerative colitis (UC) represents a debilitating condition that can lead to life-threatening complications. Current approaches for IBD treatment are far from optimal 1, 2. Therefore, alternative, safe and effective therapeutic approaches need to be developed. Regulatory T cells (Tregs), identified by the expression of the transcription factor forkhead box P3 (Foxp3), play crucial roles in the maintenance of tissue homeostasis by limiting immune responses 3, 4. Tregs can be generated in the thymus from T cell progenitors such as natural Tregs (nTregs) and from naive T cells such as induced Tregs (iTregs) 5, 6. iTregs are induced in the periphery in response to various signals, including TCR activation, cytokines (IL-2 and TGF-β1), nuclear hormone receptor ligands, and other tissue factors 7-9. Compared to nTregs, iTregs generated *in vitro* are less stable and readily transformed into effector T cells 10, 11. As a result, iTregs fail to successfully control tissue inflammation 12, 13. Although nTregs are effective at suppressing inflammation, they are difficult to harvest sufficient amounts for therapeutic applications and they can readily lose Foxp3 expression to produce IL-17 in the presence of pro-inflammatory cytokines such as IL-6 *in vivo* 14, 15. Therefore, it is highly desirable to generate iTregs with improved stability by utilizing various iTreg-inducing agents, such as rapamycin 16. Recently, a new subset of Tregs, CD4^+^CD69^+^Foxp3^+^ T cells was identified 17. It has potent immunosuppressive properties by producing sufficient amounts of IL-10 and TGF-β1 18, indicating that the generation of CD69^+^ Tregs may be a new approach to harvest Tregs with sufficient potency for the treatment of various autoimmune diseases involving Treg insufficiency. However, the regulation and clinical relevance of CD69 expression in Treg differentiation remain largely unknown.

HSF1 (heat shock transcription factor 1) is a transcription factor that regulates the expression of a battery of heat shock-related factors by binding the HSE (heat shock response element) sequence nGGAnnTTCn 19. In non-stressed cells, HSF1 is maintained in a monomeric and non-DNA binding state in the cytoplasm. Upon activation, HSF1 is trimerized and translocate to the nucleus to activate the transcription of target genes containing the HSE 20, 21. When the gastrointestinal tract is subjected to harmful stimuli, the expression of HSF1 significantly increases to protect the gastric mucosa from damages induced by harmful substances 22, 23. The HSF1 protein level is tightly regulated by ubiquitination-dependent proteasome degradation 24. Inhibition of proteasome degradation effectively stabilizes HSF1 to activate the transcription of its target genes such as HSP70 and HSP27 25. Furthermore, HSF1 can promote the expression of IL-10 in immune cells such as macrophages 26, 27. However, the relevance of HSF1 to the immunosuppressive function of Tregs remains unclear.

In this study, we found that HSF1 is important for the activation of CD69 transcription during Treg differentiation. CD69 and HSF1 deficiency resulted in much more severe colitis in mice, which could be alleviated by the transplantation of CD69^+^ Tregs but not Tregs from CD69-knockout mice. By increasing HSF1 protein levels, proteasome inhibitors (PSIs) such as MG132 and the anti-tumor drug bortezomib could promote the generation of CD69^+^ Tregs, thus offering a practical approach to generate potent iTregs for the treatment of colitis and probably other autoimmune diseases.

## Materials and Methods

### Mice and cell line

*HSF1*^+/-^ but not* HSF1*^-/-^ mice were used as mice with HSF1 deficiency because that it is difficult to have sufficient HSF1^-/-^ mice due to infertility and prenatal lethality 28, 29.* CD69*^fl/fl^ mice, *CD4*^Cre^* and Foxp3*^YFP-Cre^ mice were purchased from Cyagen Biosciences 30. Mice with various genotypes were generated by the appropriate intercrosses or backcrosses and were analyzed between 6-8 weeks of age. Female C57BL/6 mice aged 6-8 weeks were obtained from Joint Ventures Sipper BK Experimental Animal (Shanghai, China). Mice were housed in a barrier facility and all experiments were performed adhering to the guidelines and the experimental protocols approved by the Animal Care and Use Committee of the Medical School of Zhejiang University (Hangzhou, China). Human embryonic kidney cells (HEK293) were purchased from National biomedical experimental cell resource bank. All cells were cultured at 37 °C and 5% CO_2_ in DMEM medium (Gibco, USA) supplemented with penicillin (100 U/ml), streptomycin (100 mg/L), and 10% fetal bovine serum (FBS, Gibco, USA).

### Treg induction *in vitro*

Naïve CD4^+^ T cells from C57BL/6, *HSF1^+/+^*or *HSF1^+/-^* mice were negatively selected using the CD4^+^ T cell isolation Kit (Stem Cell, USA) as described previously 9. For Tregs induction, Naive CD4^+^ T cells were pretreated with different doses of proteasome inhibitor MG132 (Sigma-Aldrich, USA) or Bortezomib (Sigma-Aldrich, USA) for 2 h at 37 ℃, followed by stimulation with plate-bound 2 µg/ml anti-CD3 (eBioscience, USA), 2 µg/ml soluble anti-CD28 (eBioscience, USA), 100 IU/ml recombinant murine IL-2 (Peprotech, USA) and 10ng/ml recombinant human TGF-β1 (R&D Systems, USA) for 72 h at 37 ℃.

### RNA isolation, cDNA synthesis, and quantitative real-time PCR

Total RNA from the indicated types of cells was extracted using Trizol reagents (Invitrogen, USA) according to the manufacturers' instructions. After being quantified by NanoDrop 2000 (Nanodrop, USA), the RNA samples were reverse transcribed into cDNA using a High-Capacity cDNA Reverse Transcription Kit (Thermo Fisher, USA). The relative levels of target mRNAs were determined by qRT-PCR using the SYBR Green Master Mix Kit (Thermo Fisher, USA). The sequences of primers are listed in Table 1. Data were analyzed using β-actin as the normalization control.

### Western blot analysis and ELISA

For western blot detection, the crude proteins were extracted and the concentration of proteins was determined using a Micro BCA protein assay kit (Thermo Fisher, USA). The cell lysates (30 µg/Lane) were separated on 10-12% gels using sodium dodecyl sulfate-polyacrylamide gel electrophoresis (SDS-PAGE). The proteins in the gel were then transferred onto polyvinylidene difluoride (PVDF) membranes. After blocking with 5% fat-free dry milk in TBST, membranes were probed with primary antibodies, including anti-HSF1 (diluted 1:1000), anti-CD69 (diluted 1:500), and anti-β-actin (diluted 1:2500). The anti-HSF1 (Clone: D3L8I, Catalog: 12972S) and anti-β-actin (Clone: 8H10D10, Catalog: 3700S) antibody were purchased from Cell Signaling Technology. The anti-CD69 antibody (Clone: D-3, Catalog: sc-373799) were purchased from Santa Cruz. The bound antibodies were detected using horseradish peroxidase (HRP)-conjugated secondary antibodies (Santa Cruz, USA) and visualized by enhanced chemiluminescent reagents (Thermo Fisher, USA).

The levels of cytokines such as IL-10 and TGF-β1 in the supernatant of cultured cells were measured using ELISA kits (eBioscience, USA). For the detection of TGF-β1**,** 100 µl of supernatants were acidified with 20 µL of 1N HCl at room temperature for 10 min and then neutralized with 20 µl of 1N NaOH to activate latent TGF-β1 into its immunoreactive form.

### Tregs suppression assay

The murine splenic CD4^+^ T cells were isolated using a CD4^+^ T-cell isolation kit II (Miltenyi Biotec, Germany) and were labeled with CFSE (Invitrogen, USA) according to the manufacturer's instruction. The CFSE-labeled CD4^+^ T cells (1 × 10^6^/ml) stimulated with 1 µL of anti-CD3/CD28-coated beads (Invitrogen, USA) were served as the effector CD4^+^ T cells. Purified PSI-iTregs (Treg induced with the presence of MG132), PSI-CD69^+^ iTregs, Control-iTregs (Treg induced with the absence of MG132), and Control CD69^+^ iTregs were then added at a ratio of 1: 1, 1: 2, or 1: 4. Three days later, the cells were harvested, and the proliferation of effector CD4^+^ T cells was analyzed using flow cytometry.

### Cell staining and FACS analysis

Antibodies including APC-anti-CD4 (Invitrogen, Clone: RM4-5, Catalog: 17-0043-82) or PE-Cy7-anti-CD4 (Invitrogen, Clone: GK1.5, Catalog: 25-0041-82) and PE-anti-CD69 (Invitrogen, Clone: H1.2F3, Catalog: 12-0691-82) or PE-Cy5-anti-CD69 (Invitrogen, Clone: H1.2F3, Catalog: 15-0691-82), PE-anti-Foxp3 (Invitrogen, Clone: FJK-16s, Catalog: 12-5773-82), APC-anti-CD25 (Invitrogen, Clone: PC61.5, Catalog: 17-0251-82), PE-anti-CTLA-4 (Invitrogen, Clone: UC10-4B9, Catalog: 12-1522-82), PE-anti-ICOS (Invitrogen, Clone: C398.41, Catalog: 12-9949-81) or isotype controls(Invitrogen, USA) were used to analyze the phenotype of various Tregs. The expression levels of individual molecules in Tregs were determined using flow cytometry. Foxp3 staining was performed using the Foxp3 Fix/Perm buffer (Invitrogen, USA). After staining, cells were washed and fixed with 2% paraformaldehyde before analysis by flow cytometry. Post-analysis was performed using the FlowJo software.

### HSF1 knockdown and overexpression

*HSF1* siRNA or scrambled siRNA (Ctrl Si) were synthesized by GenePharma (Shanghai, China). The target sequences for *HSF1* are listed in Table 1. For siRNA transfection, CD4^+^ T cells (1 × 10^6^/well) were seeded in 24-well plates overnight and transfected using Lipofectamine ^TM^ RNAi MAX transfection reagent (Invitrogen, USA) for 24 h. For inhibition of HSF1, 1 × 10^6^ CD4^+^ T cells were seeded overnight in 48-well plates and 5 μM HSF1 inhibitor KRIBB11 (Sigma Aldrich, USA) were added for 24-48 h. DMSO was used as a negative control.

The HSF1 ORF (open reading frame) was amplified by PCR and inserted into a lentivirus vector (pPCDH-CMV-MCS-EF1-GFP) after sequencing confirmation**.** CD4^+^ T cells were then transfected with the packaged recombinant lentivirus or scrambled control vector. Briefly, viral supernatant was concentrated using PEG-it virus precipitation solution (System Biosciences, USA). High titer virus was then incubated with isolated CD4^+^T cells, followed by spinoculated for 2 h at 300g with polybrene (8 mg/ml) and cultured for 48 h. The expression of CD69 after transduction was confirmed by using FACS.

### Chromatin immunoprecipitation (ChIP)

The interaction of HSF1 with CD69 promoter in CD4^+^ T cells was characterized by ChIP assays according to the manufacturer's protocol (Millipore, USA). Briefly, CD4^+^ T cells stimulated with or without anti-CD3/CD28 or pretreated with 10 µM MG132 for 2 h were cultured under iTreg polarization condition for 24 h. Cells were then fixed with 1% formaldehyde for 10 min at 37 °C. After sonication, the recovered DNA fragments were incubated with antibodies overnight at 4 °C. The antibodies used were anti-HSF1 (Abcam, Clone: EP1710Y, Catalog: ab52757) and rabbit IgG antibodies (Cell Signaling Technology, Clone: DA1E, Catalog: 3900). Abs/chromatin complexes were precipitated using protein G agarose (Millipore, USA) and then reversed the cross-linking at 65 °C for 4 h. For IP samples, protein G magnetic beads were separated by a magnetic separator rack, and supernatant was used for DNA purification. The presence of indicated DNA sequences were assessed using qRT-PCR with primers listed in Table 1.

### Luciferase reporter assay

The promoter of mouse HSF1 and its truncations were produced by PCR-based amplification and subcloned into the pGL3-Enhancer vector to form luciferase reporter plasmids. HEK293 cells were cotransfected with 100 ng of the luciferase reporter plasmid, 10 ng of the thymidine kinase promoter-Renilla luciferase reporter plasmid plus pCMV-HSF1 or the control vector. After 48 h, luciferase activity was determined using the Dual-Luciferase Reporter Assay System (Promega, USA) according to the manufacturer's instructions.

### Cell Preparation

Lamina propria (LP) cells in the colon were isolated by a modified method described previously 31. In brief, the gut pieces were cut into approximately 2-mm slices and the epithelium was removed by stirring, first in PBS containing 3 mM EDTA for 10 min at 37 ∘C and then in RPMI 1640 containing 2% FBS, 1 mM EGTA, and 1.5 mM MgCl_2_ for 15 min. Gut pieces were collected and stirred in RPMI 1640 containing 20% FBS, 100 U/ml collagenase (Sigma-Aldrich, USA), and 5 U/ml DNase 1 (Sigma-Aldrich, USA) for 120 min at 37 ℃. The pellet was purified to LPL on a 45%/66.6% discontinuous Percoll (GE Healthcare, USA) gradient at 800 × g for 30 min. The number of viable cells was counted by trypan blue staining.

### Induction and treatment of intestinal colitis in mice

Colitis was induced in female C57BL/6, *HSF1*^+/+^, *HSF1*^+/-^,* CD4*^Cre^*CD69*^fl/fl^, and *Foxp3*^YFP-Cre^*CD69*^fl/fl^ mice at 6 weeks of age. Mice were first randomized and administered with 2% (w/v) of dextran sulfate sodium (DSS, 40 kDa, MP Biomedicals, USA) in drinking water for 5-10 days. Control mice received normal drinking water. The body weights of mice were monitored daily. For KRIBB11 inhibition, mice were pretreated with a single dose of KRIBB11 (100 μl, 5 mg/kg) 1 h before DSS drinking. For proteasome inhibitor treatment, mice were injected intraperitoneally 15 μm/kg MG132 or 350μg/kg Bortezomib dissolved in DMSO for 9 days. For cells transplantation, mice two of days post-DSS-induction were injected intravenously with purified PSI-iTregs or Control-iTregs (1 × 10^6^ cells/mouse, purity of the cells>95%). Body weight change and disease activity index (DAI) were recorded from day 1 to the end of the study. The DAI was calculated as the sum of weight loss, the rigidity of stool specimens, and the extent of hematochezia. At the end of the experiment, the large intestines of individual mice were dissected out and fixed in 10% phosphate-buffered formalin. Fixed samples were processed by paraffin embedding, sectioning, and hematoxylin and eosin (H & E) as standard procedures.

## Results

### CD69 is important for maintaining potent Treg function to inhibit colitis

**Figure 1:**
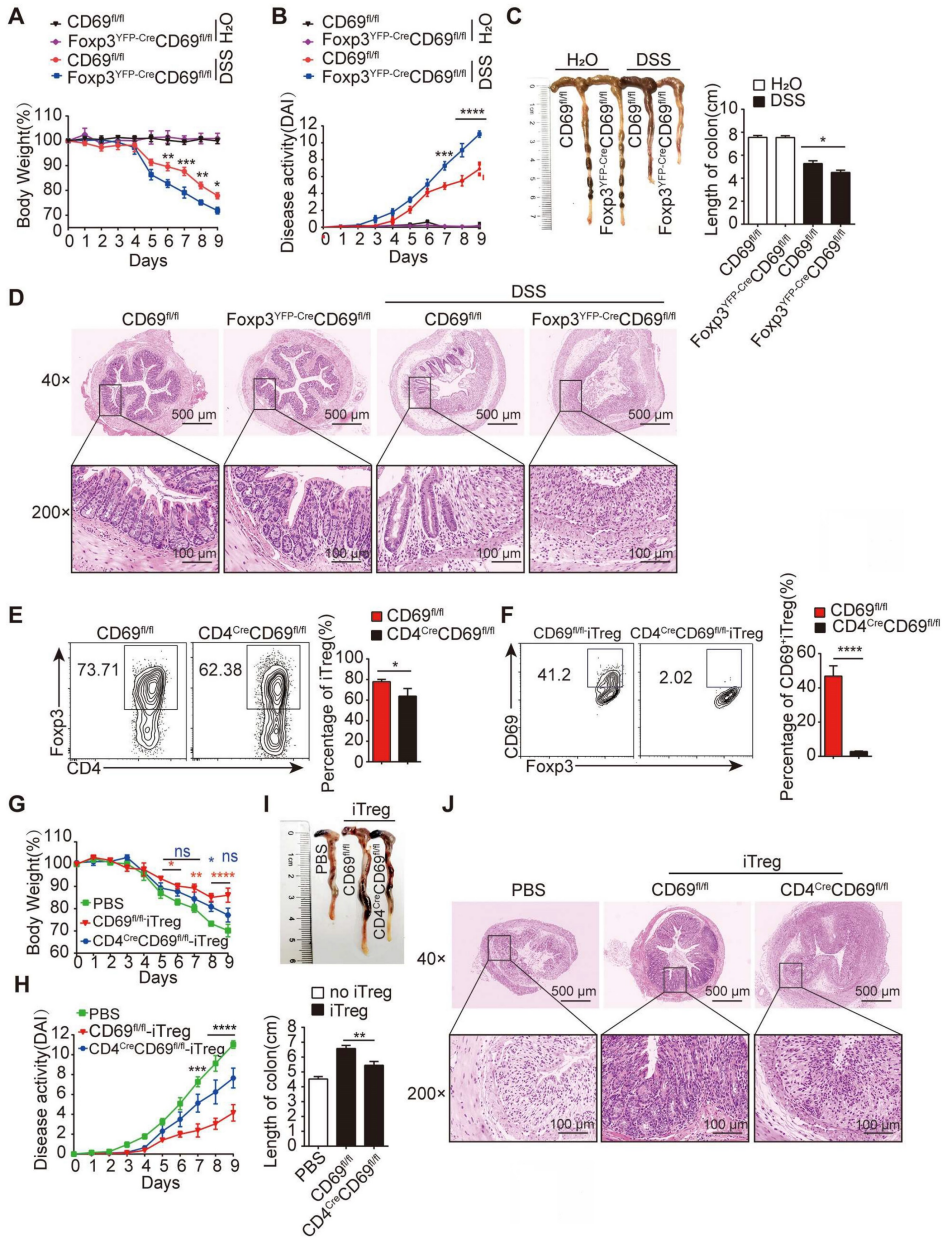
** CD69 is indispensable to maintain iTregs functio**n. (**A**) *CD69*^fl/fl^ or *Foxp3*^YFP-Cre^*CD69*^fl/fl^ mice were administrated with 2% DSS in drinking water for 9 days (n = 7 for each group). The average body weight is shown as a percentage relative to the initial value. (**B**) The disease activity index is analyzed. The length of the colon (**C**) and histological appearance (**D**) after 9 days of colitis induction. (**E and F**) Naïve CD4^+^ T cells isolated from *CD69*^fl/fl^ or* CD4*^Cr*e*^*CD69*^fl/fl^ mice were cultured for 3 days under Treg-polarization conditions and then analyzed by flow cytometry. *Foxp3*^YFP-Cre^*CD69*^fl/fl^ mice after 2 days of DSS administration were intravenously injected with iTregs from *CD69*^fl/fl^ and *CD4*^Cr*e*^*CD69*^fl/fl^ mice (n = 7 for each group). The mice's body weight (**G**)and the DAI (**H**) were recorded every day. The average body weight is shown as a percentage relative to the initial value. (**I**)The average length of the colons. (**J**) Hematoxylin and eosin staining of the colon sections. Scale bar:500 μm (whole colon section) and 100 μm (enlarged insets). ns, not significant. *p < 0.05, **p < 0.01, ***p < 0.001, ****p < 0.0001 compared with the control or the indicated group.

To clarify the role of CD69^+^ Tregs in colitis, we generated a conditional *CD69* allele in which exons 2 to 4 were flanked by loxP sites, and *Foxp3*^YFP-Cre^*CD69*^fl/fl^ or *CD4*^Cre^*CD69*^fl/fl^ mice were generated to conditionally knock out CD69 in Foxp3^+^ or CD4^+^ T cells by crossing *CD69*^fl/fl^ mice with *Foxp3*^YFP-Cre^ or *CD4*^Cre^ mice (Figure S1A-B). Considerably, fewer CD69^+^ Tregs were detected in *CD4*^Cre^*CD69*^fl/fl^ and *Foxp3*^YFP-Cre^*CD69*^fl/fl^ mice (Figure S1C-D), confirming the successful construction of conditional CD69 knockout mice. Consistent with the previous *in vitro* results 18, the expression levels of IL-10 and TGF-β1 were reduced in CD69-deficient Tregs (Figure S1E). Next, we compared the time course of colitis development in *Foxp3*^YFP-Cre^*CD69*^fl/fl^ mice versus their control littermates (*CD69*^fl/fl^) by monitoring body weight and the disease activity index (DAI). Following DSS administration for 9 days, body weight loss was significantly more pronounced in *Foxp3*^YFP-Cre^*CD69*^fl/fl^ mice compared to that in* CD69*^fl/fl^ mice (Figure 1A). The symptoms and disease activity were much severe and colon length was much shorter in *Foxp3*^YFP-Cre^*CD69*^fl/fl^ mice (Figure 1B-C), which was accompanied by more damaged of the crypt architecture and increased infiltration of inflammatory cells in the colon of *Foxp3*^YFP-Cre^*CD69*^fl/fl^ mice (Figure 1D). In addition, colitis was successfully induced only in *Foxp3*^YFP-Cre^*CD69*^fl/fl^ but not *CD69*^fl/fl^ mice when DSS was administered for 5 days followed by normal water (Figure S1F-G), highlighting the importance of CD69^+^ Tregs in the inhibition of colitis.

To further confirm the relevance of CD69^+^ Tregs in colitis, we generated iTregs under Treg polarization conditions *in vitro*. While the generation of general iTregs (CD4^+^Foxp3^+^) was slightly impaired, naive CD4^+^ T cells of *CD4*^Cre^*CD69*^fl/fl^ but not* CD69*^fl/fl^ animals failed to differentiate into CD69^+^ iTregs in the presence of exogenous IL-2 and TGF-β1 *in vitro* (Figure 1E-F). Tregs induced by *CD69*^fl/fl^ but not *CD4*^Cre^*CD69*^fl/fl^ mice secreted sufficient IL-10 and TGF-β1 to inhibit the proliferation of co-cultured CD4^+^ T cells (Figure S1H-I). Furthermore, mice receiving with iTregs from *CD69*^fl/fl^ mice but not *CD4*^Cre^*CD69*^fl/fl^ mice, showed a significant improvement in weight loss (Figure 1G). Consistent with this, the disease activity was also reduced, and the colon length decrease was reversed by iTregs from *CD69*^fl/fl^ mice, but not from *CD4*^Cre^*CD69*^fl/fl^ mice (Figure 1H-I), accompanied by alleviation of leukocytes infiltration and damage to the mucosal layer of the colon (Figure 1J). Taken together, CD69 is critical for the ability of iTregs to inhibit colitis.

### HSF1 is important for CD69 transcription in CD4^+^ T cells

**Figure 2:**
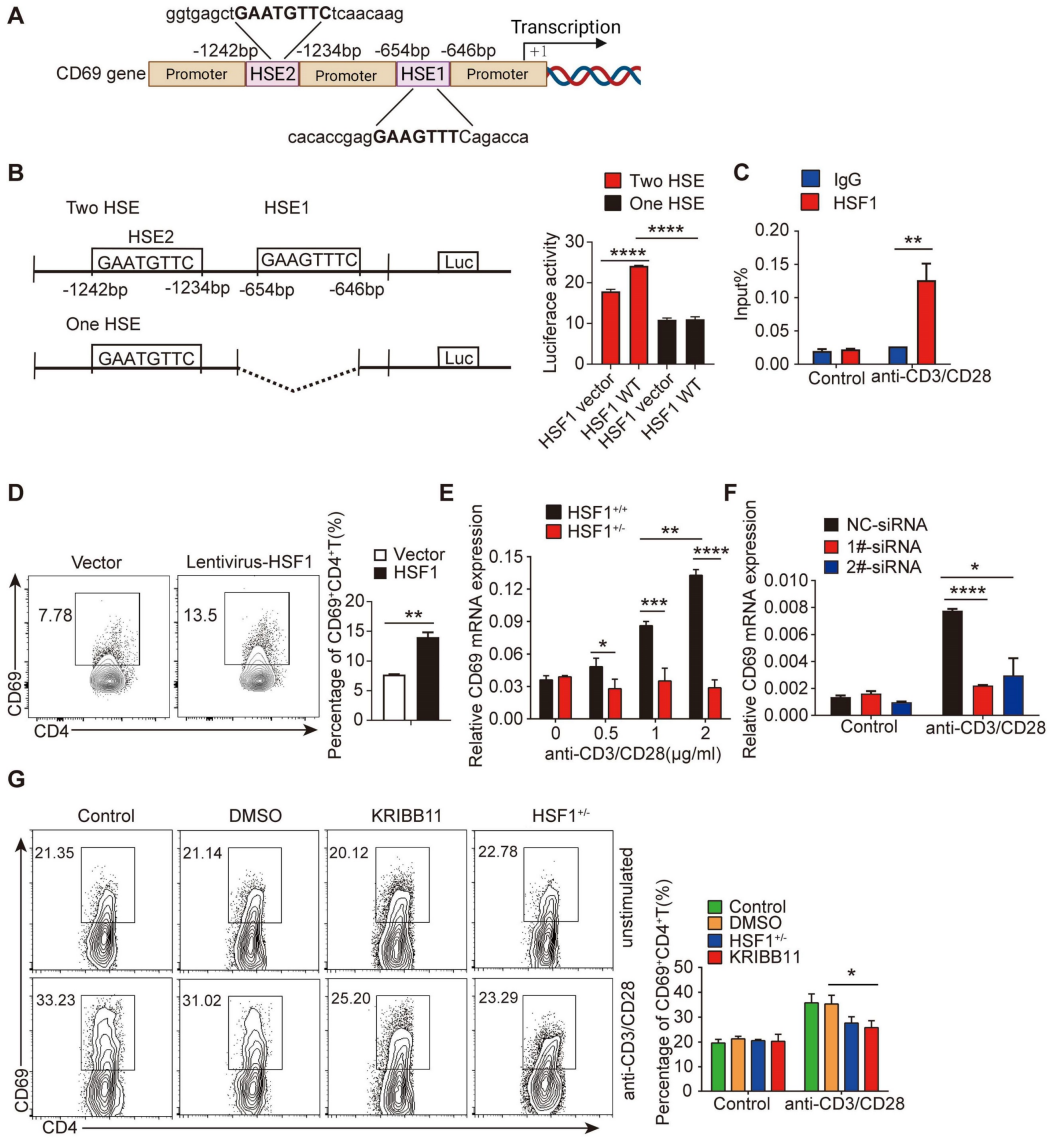
**HSF1 is important for CD69 transcription in CD4^+^ T cells.** (**A**) Schematic of the nucleotide sequence of CD69 promoter, indicating the locations of the putative HSE sites. The potential HSF1 binding sites are shown in boxes. (**B**) HEK293 cells were infected for 48 h with luciferase reporter plasmids as shown on the left as well as a construct that encodes HSF1 or the empty vector, and then, cells were harvested to detect luciferase activity. (**C**) ChIP analysis of HSF1 binding to the CD69 promoter in CD4^+^ T cells with or without anti-CD3/CD28 mAb stimulation. (**D**) Overall 1×10^6^/mL CD4^+^ T cells were added to 8 μg polybrene and transfected with control lentivirus or lentivirus-HSF1 at an MOI of 100 for 48 h. CD69 protein levels were determined using flow cytometry. (**E**) CD4^+^ T cells isolated from *HSF1^+/+^* and *HSF1^+/-^* mice were stimulated with different doses of anti-CD3/CD28 antibodies, and the mRNA levels of CD69 were detected by qRT-PCR. (**F**) 1 × 10^6^/mL CD4^+^ T cells were stimulated with or without anti-CD3/CD28 antibodies and treated with *HSF1* siRNA for the indicated time. The relative levels of CD69 expression in different groups of CD4^+^ T cells were determined by qRT-PCR. (**G**) CD4^+^ T cells isolated from *HSF1^+/+^* and *HSF1^+/-^* mice were stimulated with or without anti-CD3/CD28 antibodies following the treatment with KRIBB11 for 24 h, and then the expression of CD69^+^CD4^+^ T cell was analyzed by FACS. Representative images of the data expressed as mean ± SD of three independent experiments (n = 7 per group). ns, not significant. *p <0.05, **p < 0.01, ***p < 0.001, ****p < 0.0001, as analyzed by ANOVA or Student's *t*-test.

As previously reported, CD69 mRNA was increased upon Treg differentiation and the engagement of the T cell receptor/CD3 complex 32, 33. Notably, typical heat shock response elements (HSE) characterized by an array of nGAAnnTTCn motifs, were found in the promoter region of the CD69 (Figure 2A), hinting that CD69 transcription could be regulated by HSF1 34. Indeed, HSF1 succeeded to increase the activity of the luciferase driven by *CD69* promoter containing intact HSE (Figure 2B). To investigate whether HSF1 was recruited to CD69 promoter, CD4^+^ T cells were treated with or without anti-CD3/CD28 antibodies for 24 h, and HSF1 bound DNA was then analyzed using qRT-PCR with specific primers amplifying the CD69 promoter region containing HSE. Consistent with the result of luciferase activity assay, HSF1 was recruited to the CD69 promoter in activated CD4^+^ T cells (Figure 2C). To explore the relevance of HSF1 to promote CD69 production in CD4^+^ T cells, we introduced exogenous HSF1 to CD4^+^ T cells by infecting them with the lentivirus with the HSF1-expressing construct and found CD69 expression was indeed significantly increased (Figure 2D). After stimulation with anti-CD3/CD28 antibodies, CD69 mRNA was significantly up-regulated in a concentration-dependent manner in CD4^+^ T cells of *HSF1*^+/+^ but no*t HSF1*^+/-^ mice (Figure 2E). However, such upregulation of *CD69* mRNA were compromised after the knockdown of *HSF1* expression with siRNA (Figure 2F). As a result, CD69^+^ T cells generation was greatly impaired once HSF1 activity was inhibited by its chemical inhibitor KRIBB11, or HSF1 expression was reduced by genetic knockout (HSF1^+/-^ mice) (Figure 2G and Figure S2A). Taken together, HSF1 is essential for the efficient transcription of *CD69*.

### HSF1 promotes CD69^+^ Treg differentiation to repress colitis

**Figure 3:**
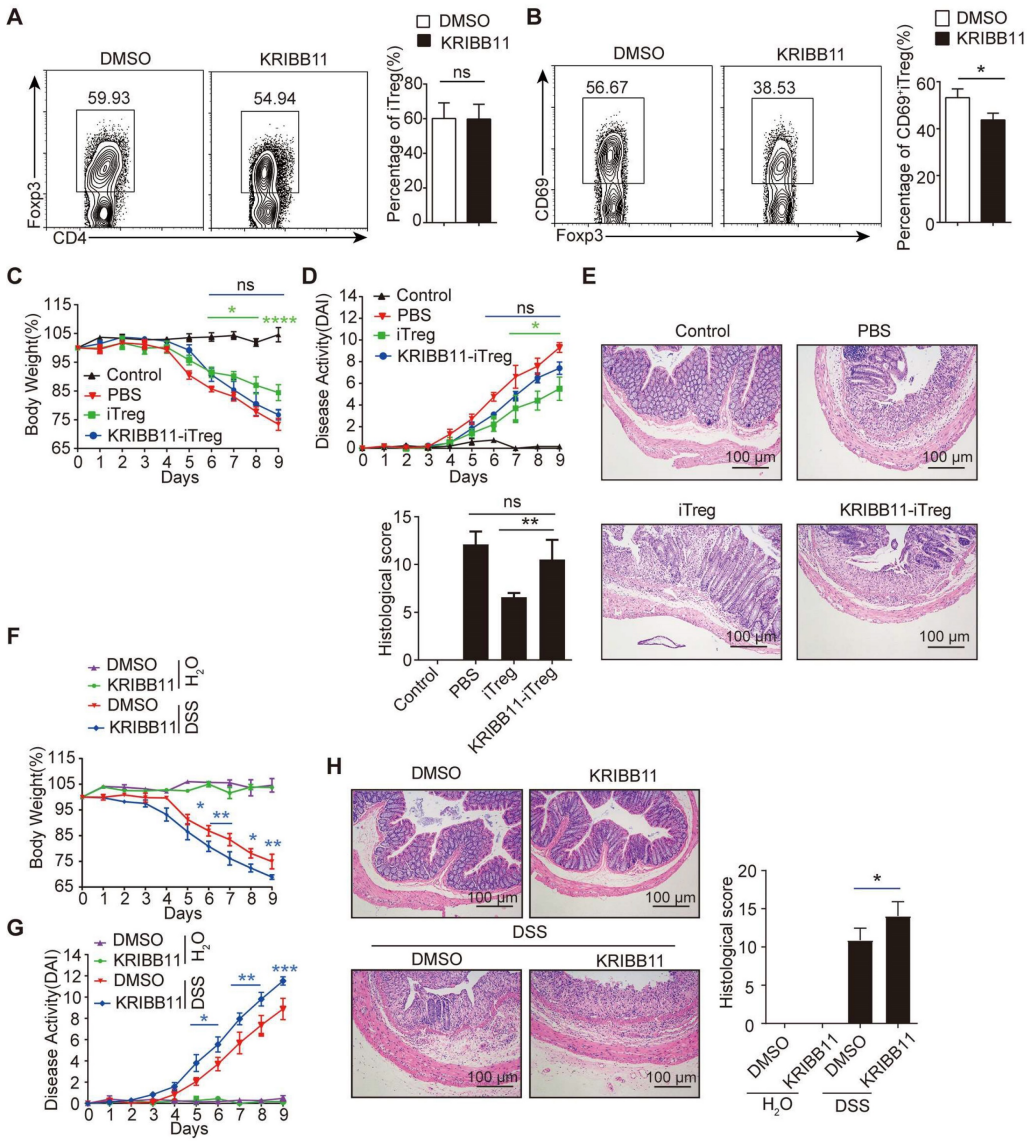
** HSF1 is necessary for CD69^+^ Treg differentiation.** Naive CD4^+^ T cells were cultured in Treg polarization conditions with or without KRIBB11. The frequency of iTreg (**A**) and CD69^+^iTreg (**B**) was analyzed by FACS. Induced Tregs were sorted and injected i.v into IBD mice treated with KRIBB11 on day 2. The body weights (**C**) were measured and DAI (**D**) was analyzed daily. (**E**) H&E-stained images of colon sections. Scale bar: 100 μm. (**F-G**) The mice were treated with KRIBB11 and then administration with 2% DSS, body weight and DAI were analyzed daily. (**H**) H&E-stained images of colon sections. Scale bar: 100 μm. Representative images of the data expressed as mean ± SD of three independent experiments (n = 7 per group). ns, not significant. *p < 0.05, **p < 0.01, ***p < 0.001, ****p < 0.0001 as analyzed by ANOVA and Student's *t*-test.

To explore the relevance of HSF1 in CD69^+^ Treg differentiation, iTregs were induced in naive CD4^+^ T cells from *HSF1^+/-^* mice or* HSF1^+/+^* mice in the presence of the HSF1 inhibitor KRIBB11. CD69^+^iTreg differentiation was impaired in CD4^+^ T cells with HSF1 deficiency, although general iTregs differentiation was not affected (Figure 3A-B). The iTregs induced in the presence of KRIBB11 failed to improve colitis once transferred into mice with colitis (Figure 3C-E). Consequently, KRIBB11 exacerbated DSS-induced colitis in mice. After inhibition of HSF1, more weight loss was observed in mice with DSS-induced colitis (Figure 3F), which coincided with increased disease activity and tissue injury (Figure 3G-H). Moreover, the frequency of CD69^+^ Tregs was also compromised in mice treated with the HSF1 inhibitor KRIBB11 (Figure S3A). In summary, these results indicate that HSF1 is important for differentiation of CD69^+^ Tregs with potent immunosuppressive functions.

### Proteasome inhibitors stabilize HSF1 protein to promote CD69^+^ Treg differentiation

**Figure 4:**
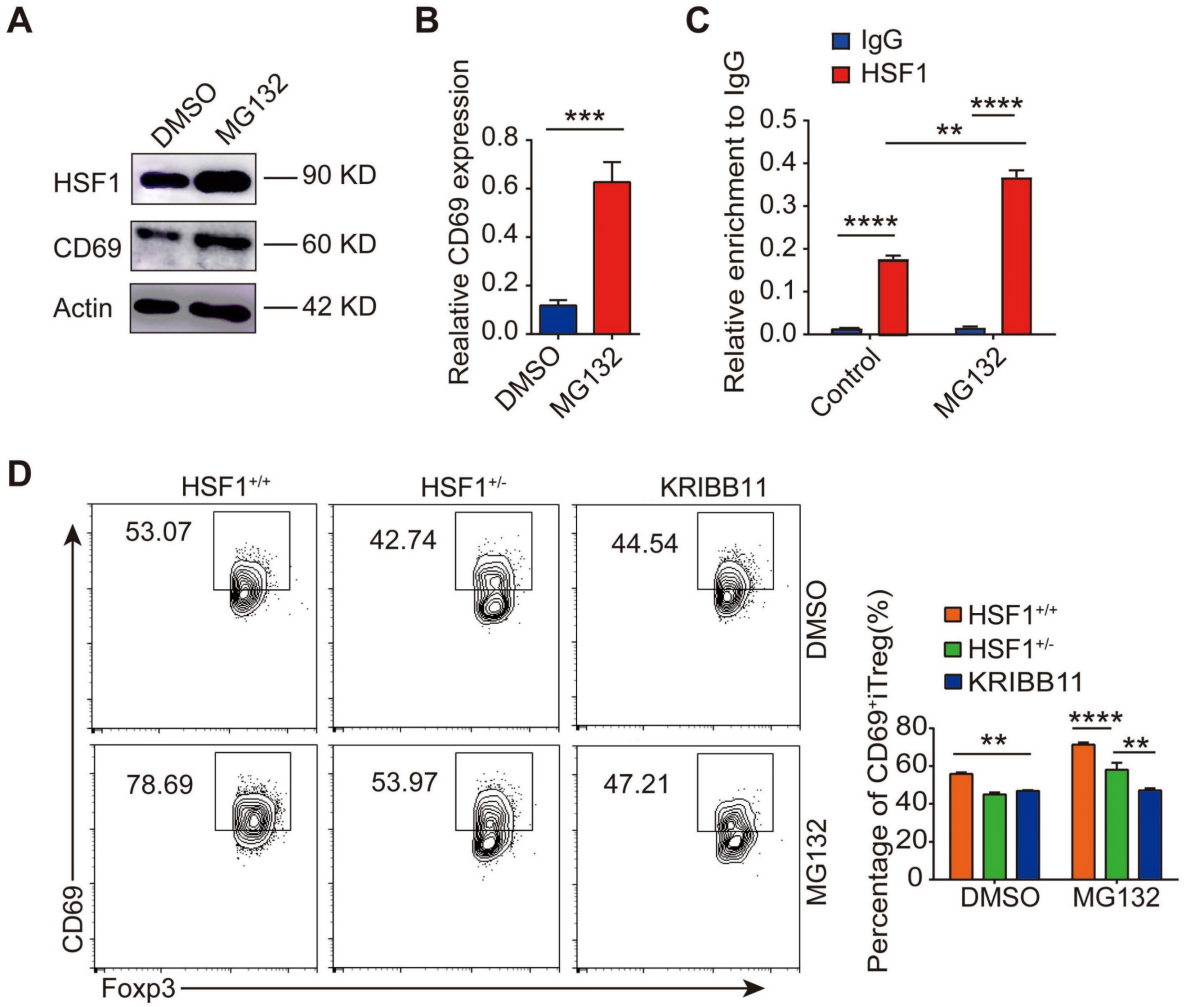
** MG132 prompts the activation of HSF1 and the expression of CD69.** 1 × 10^7^ CD4^+^ T cells were isolated and incubated with 10 µM of MG132 at 37°C for 2 h and then cultured in T lymphocyte culture medium for 24 h. (**A**) All the cells were harvested and protein levels of HSF1 and CD69 were analyzed by western blot. (**B**) The relative levels of *CD69* were analyzed by qRT-PCR. (**C**) CD4^+^ T cells were incubated with MG132 for 2 h and then washed with 1640 RPMI, followed by culture under Treg polarization for 24 h. Cells were then subjected to a ChIP assay using the indicated antibodies. The precipitated DNA was analyzed by quantitative PCR using primer pairs corresponding to the indicated genomic regions. (**D**) CD4^+^ naive T cells were sorted from *HSF1*^+/+^ o*r HSF1*^+/-^ mice and pretreated with MG132 and induced iTregs while HSF1 inhibitor was added at the same time to block HSF1 expression. The flow cytometric analysis of iTregs and CD69^+^ iTregs expression. Representative images of the data expressed as mean ± SD of three independent experiments. **p < 0.01, ***p < 0.001, ****p < 0.0001. ns, not significant, as analyzed by ANOVA or Student's *t*-test.

As HSF1 protein abundance is regulated by proteasomal degradation, its expression was increased upon inhibition of proteasomal degradation by MG132 (Figure 4A), thus activating the transcription of its downstream target genes such as *HSP90AB1*, *DNAJB1*, and *HSPA1A* (Figure S4A). Strikingly, the expression of both CD69 protein and mRNA was increased in CD4^+^ T cells treated with the proteasome inhibitor MG132 under Treg polarization condition (Figure 4A-B), accompanied by increased interaction of HSF1 with the *CD69* promoter (Figure 4C and Figure S4B). However, the turnover of CD69 protein was most likely unaffected by MG132 (Figure S4C). Moreover, inhibition of proteasome degradation by MG132 promoted the generation of CD69^+^ iTregs, which was impaired when HSF1 was either chemically inhibited or genetically knocked out (Figure 4D). Therefore, inhibition of proteasomal degradation stabilizes HSF1 protein to promote CD69^+^ Tregs differentiation.

### Proteasome inhibitors promote CD69^+^ Tregs differentiation *in vivo* but fail to alleviate colitis effectively

**Figure 5:**
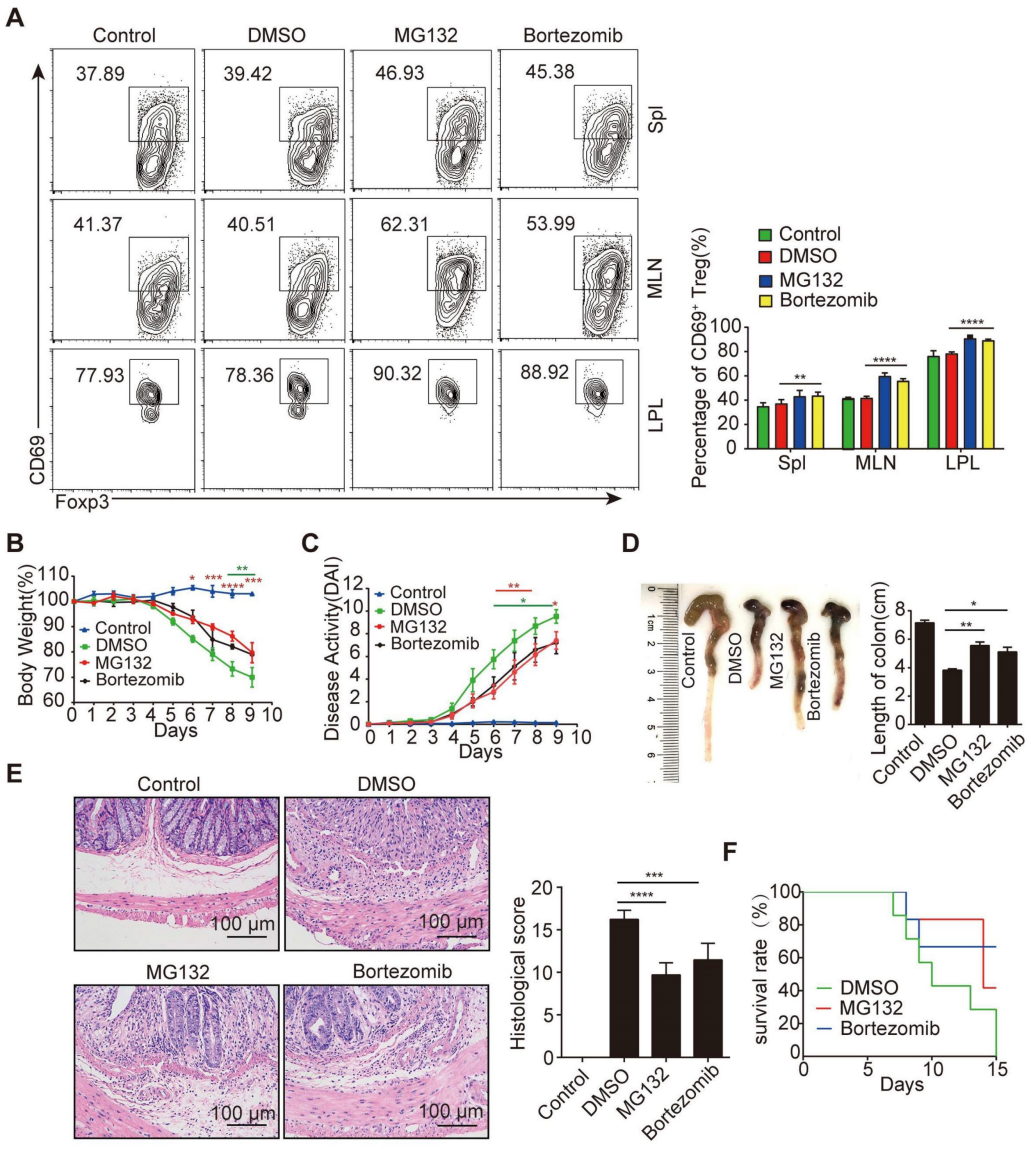
** Proteasome inhibitors showed a milder therapeutic effect on IBD.** (**A**) Density plots showing CD69 expression in gated CD4^+^Foxp3^+^ cells from freshly isolated spleen, MLN and colonic LPL in mice treated with MG132 and bortezomib. Acute colitis was induced in animals by administering 2% DSS in their drinking water for 9 days. Mice were treated with or without MG132 and bortezomib for 2 days at indicated doses. Changes in body weight (**B**), disease activity index (**C**), and colon length (**D**) and histological sections of inflamed colons (**E**) during the course of DSS treatment in each group of mice. Scale bar:100 μm. (**F**) Survival rates of each group of mice after the initiation of DSS-induced acute colitis were recorded daily (n=10 per group). Representative images of the data expressed as mean ± SD of three independent experiments. *p < 0.01, **p < 0.01, ***p < 0.001, ****p < 0. 0001.ns, not significant, as analyzed by ANOVA or Student's *t*-test.

To confirm the effect of proteasomal degradation inhibition on Treg differentiation *in vivo*, we used proteasome inhibitors (PSIs) including MG132 and bortezomib, a drug clinically applied for the treatment of myeloma. Indeed, inhibition of proteasomal degradation by PSIs increased the frequency of CD69^+^ Tregs (Figure 5A) but not the frequency of CD4^+^Foxp3^+^ Tregs (Figure S5A). After DSS treatment, mice administered PSIs exhibited less weight loss (Figure 5B) and showed a reduction in the overall disease activity index, as well as colon length-shortening (Figure 5C-5D). Although a reduced histopathological score was observed in the colon tissues, disrupted crypt architecture and inflammatory cell infiltration were still evident (Figure 5E). Moreover, in difference in survival was observed in mice with or without PSIs treatment (Figure 5F). These results suggested that PSIs can promote CD69^+^ Treg differentiation but fail to effectively protect mice from colitis.

### PSIs promote CD69^+^ iTreg differentiation* in vitro*

**Figure 6:**
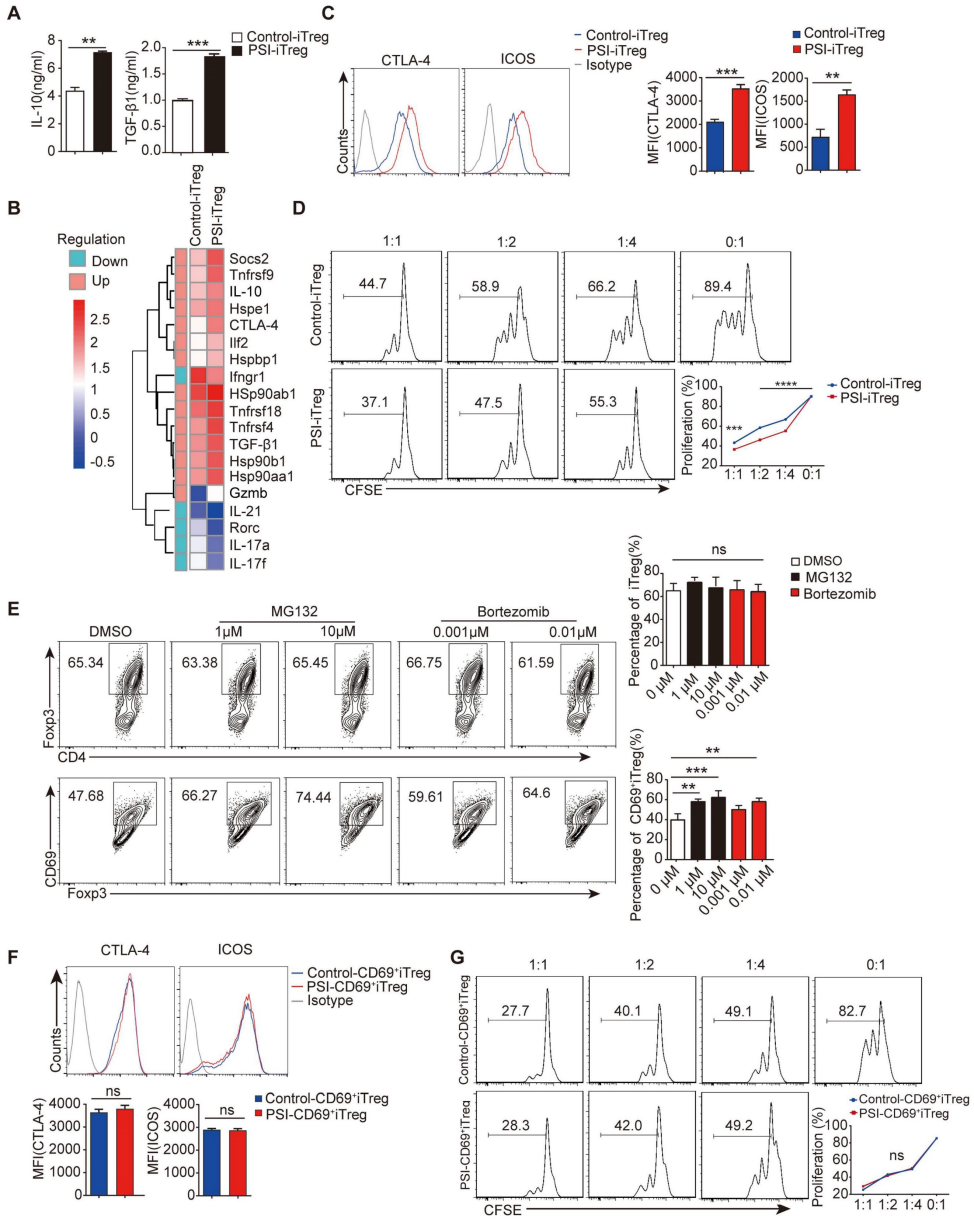
** Proteasome inhibitors prompt CD69 expression *in vitro*.** Overall 1 × 10^6^/ml freshly isolated naive CD4^+^ T cells incubated with or without MG132 for 2 h at 37°C and washed three times with 1640 RPMI. The cells were then stimulated with 2 µg/ml anti-CD3/CD28 antibody, 10ng/ml TGF-β1, and 50 IU/ml of IL-2 for 72 h. (**A**) The levels of IL-10 and TGF-β1 in the supernatants of cultured cells were measured by ELISA. (**B**) Total RNA was extracted from isolated PSI-iTregs and Control-iTregs while the gene expression profile was analyzed using the microarray analysis. The heat map of the gene expression is also shown. (**C and F**) The expression levels of CTLA-4 and ICOS in PSI-iTregs/CD69^+^iTregs and Control-iTregs/CD69^+^ iTregs were analyzed using flow cytometry with indicated antibodies. (**D and G**) 1 × 10^6^/ml of CD4^+^CD25^-^ T cells were labeled with CFSE and co-cultured with PSI-iTregs/CD69^+^ iTregs and Control-iTregs/CD69^+^ iTregs at a ratio of 1:1, 1: 2, or 1: 4 in the presence of 1 µl anti-CD3/CD28 coated beads for 3 days. The proliferation of CD4^+^ T cells was analyzed by flow cytometry. The cells were first gated on living lymphocytes and then on CFSE^+^ T cells (n = 3). (**E**) Naive CD4^+^ T cells incubated with or without MG132 or Bortezomib then cultured under Treg porlarization condition, the relative frequency of iTregs and CD69^+^ iTregs was analyzed using FACS (n = 3). Representative images of the data were expressed as mean ± SD of three independent experiments while Student's *t*-test was used for statistical analysis. *p < 0.01, **p < 0.01, ***p < 0.001, ****p < 0.0001. ns, not significant.

Tregs induced *in vitro* are intrinsically unstable to maintain immunosuppressive potency when transplanted *in vivo* 35, thus urging novel approaches to generate stable and potent iTregs with sufficient amounts. Interestingly, we found that PSI significantly enhanced IL-10 and TGF-β1 production of iTregs *in vitro* (Figure 6A). Significantly higher levels of IL-10 and TGF-β1 were detected in PSI-iTregs than in their levels from Control-iTregs. Gene expression profiling further confirmed that PSI-iTregs expressed higher levels of immuno-inhibitory genes, whereas the expression of pro-inflammation genes was downregulated (Figure 6B). Immunosuppression-related markers, such as CTLA-4 and ICOS, were also higher in PSI-iTregs than in Control-iTregs (Figure 6C). PSI-iTregs displayed a more pronounced inhibitory effect on T cells proliferation (Figure 6D). Therefore, Treg function was enhanced by proteasome inhibition, which promoted CD69^+^ iTregs differentiation. Indeed, PSIs increased CD69^+^ iTregs frequency in a dose-dependent manner, whereas the frequency of general Tregs was not affected (Figure 6E). Moreover, there was no difference in the expression of immunosuppression-related markers, and the immune inhibitory function of CD69^+^iTregs with or without PSIs (Figure 6F-G). In summary, PSIs promotes the *in vitro* differentiation of CD69^+^ iTregs to enhance the immune suppressive function of iTreg, thus representing a novel approach to obtain sufficient iTregs for colitis therapy.

### Adoptive transfer of PSI-iTregs attenuated the severity of DSS-induced IBD in mice

**Figure 7:**
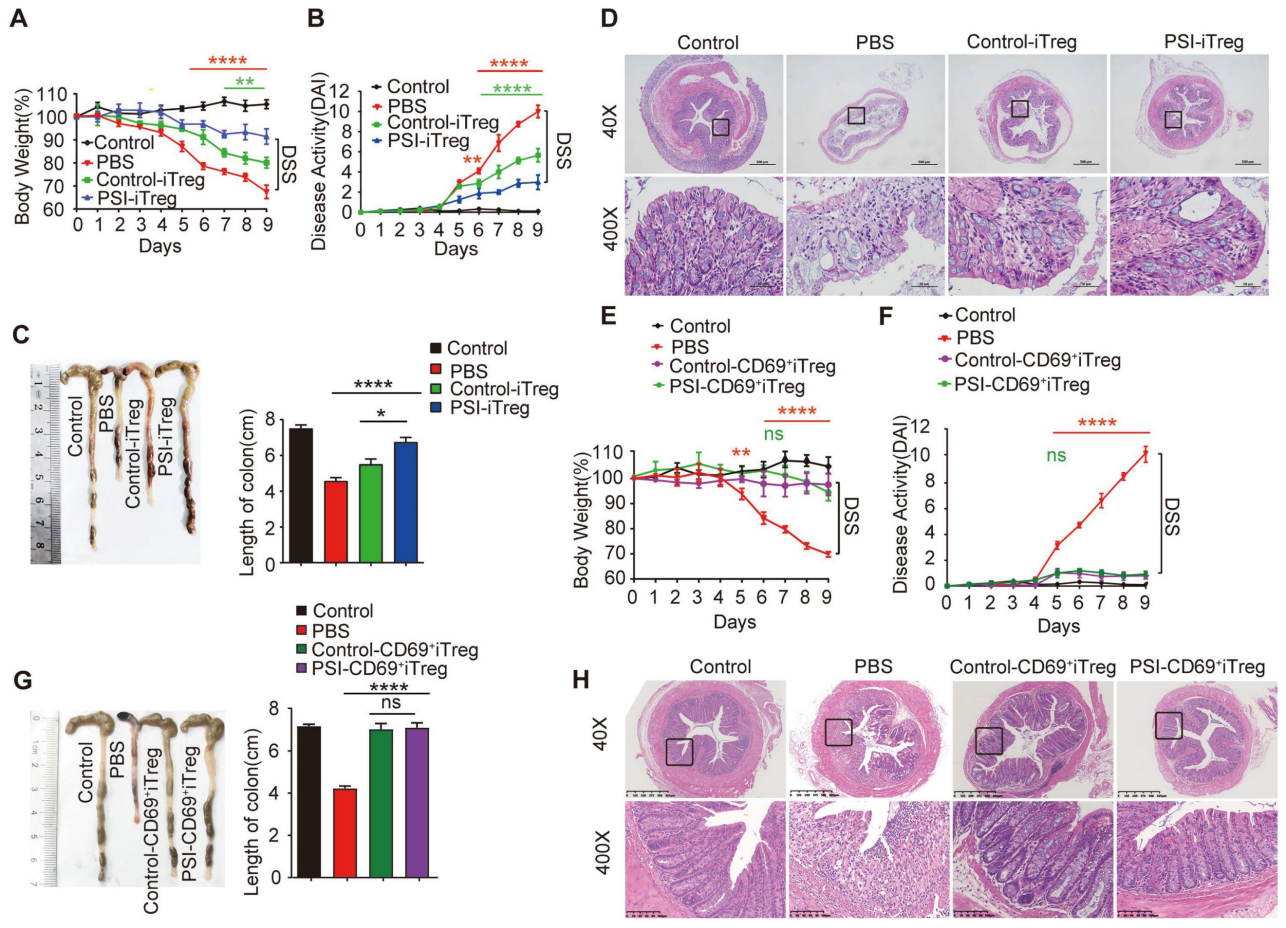
** Adoptive transfer of PSI-iTregs attenuated the severity of DSS-induced IBD in mice.** IBD was induced by administering DSS in drinking water for 10 days. For the treatment of IBD, 1 × 10^6^ PSI-iTregs or Control-iTregs were sorted and intravenously injected into mice on day 2. (**A**) The loss in body weight was recorded daily. Each point represents the average weight data pooled from eight mice ± SD. Control group: the mice fed with normal water; PBS group, the mice were drinking water containing 2% DSS and intravenously treated with PBS on day 2. (**B**) The DAI was evaluated daily. Each point represents the average DAI data pooled from eight mice. (**C**) Appearance and statistical analysis of colon length on day 9. (**D**) Representative H&E staining of the colon mice of different groups. (**E-F**) Weight loss and DAI in mice transferred with PSI-CD69^+^iTregs and Control-CD69^+^iTregs. (**G-H**) The length of colon and H&E staining of the colon from mice from the different groups. Representative images of the data expressed as the mean ± SD of three independent experiments. * *p* < 0.05, **p < 0.01, ***p < 0.001, ****p < 0.001, ns, not significant. ANOVA or Student's* t*-test was used to determine the significance.

To further confirm that the immune suppressive function of iTregs recovered from proteasomal inhibition, we compared the effects of these iTregs on colitis in mice. After transferring iTregs to mice with colitis, colitis-induced symptoms and histological changes, including body weight loss, diarrhea, and rectal bleeding, were significantly alleviated in the PSI-iTregs-treated mice compared to those in the Control-iTregs-treated littermates (Figure 7A-D). Similar to the results of the *in vitro* experiments, the sorted CD69^+^ iTregs from Control-iTregs or PSI-iTregs exerted similar effects on the colitis treatment (Figure 7E-H). Consistent with this, the frequency of Th1 and Th17 cells in mice treated with DSS was increased, which was compromised by treatment with PSI-iTregs (Figure S6A-C). Furthermore, the production of pro-inflammatory such as IFN-γ, IL-17 and IL-6 production was lower in mice treated with PSI-iTreg than in mice treated with Control-iTregs (Figure S6D).

To further confirm the value of PSI-iTregs in the treatment of IBD, we applied another model of colitis, T cell transfer-induced colitis of *Rag1*^-/-^ mice. Similar to chemically induced IBD, T-cell transfer-induced colitis in *Rag1*^-/-^ mice was also significantly attenuated by PSI-iTregs while mice treated with Control-iTregs had much more damage, with more leukocyte infiltration (Figure S7). In summary, proteasomal inhibition promotes the generation of CD69^+^ iTregs to increase the immunosuppressive potency of iTregs.

## Discussion

CD69 is much more than a simple activation marker of leukocytes, as it is an important regulator of immune responses, depending on the type of cells 36. CD69-expressing CD4^+^ T cells have powerful immunosuppressive properties. In different murine disease models including asthma, arthritis, myocarditis, pathogen clearance, the absence of CD69 expression deeply affects the disease course by exacerbating the disease severity in most cases 37-39. On the other hand, the pro-inflammatory and pathogenic roles of CD69 have been suggested by several observations. For example, CD69-expressing T-cells from the inflamed regions of patients with rheumatoid arthritis induce macrophages to secrete TNF-α 40, 41. In CD69-deficient mice, OVA-induced eosinophilic airway inflammation, mucus hyperproduction, and airway hyper-responsiveness were attenuated 42. Therefore, CD69 might be a double-edged sword for immunosuppressive function due to the complexity of host cells and CD69 may induce different signaling pathways in different types of cells. Nevertheless, CD69 seems to be critical for the immunosuppressive potency of Tregs to inhibit the development of colitis. Therefore, adoptive transfer of CD69^+^ iTreg cells may be a reasonable approach as a novel cell therapy for IBD.

Although it is expressed upon activation in many of leukocytes, CD69 is predominantly expressed on T cells. Different types of activators, including type I IFN, phorbol esters, PMA, LPS and anti-CD3 antibodies, are known to rapidly induce CD69 expression 43, 44. Expression of the CD69 gene can be regulated by several transcriptional factors, including activator protein-1 (AP-1), nuclear factor (NF)-κB, and erythroblast transformation-specific related gene-1 (ERG-1) 45, 46. In this study, we showed that HSF1 activated the transcription of CD69 to promote CD69^+^ Treg differentiation *in vitro* and *in vivo* (Figure 7E). As the results, HSF1 is important to the function of Treg and stabilization of HSF1 promotes the differentiation of CD69^+^ Tregs. Interestingly, we did find that inhibition of HSF1 affected cell activation using CD25 as the activation marker, although CD69 expression was further reduced (data not shown). In addition, ectopic HSF1 expression up-regulated CD69 expression even in the absence of T cell activation by anti-CD3/CD28 antibodies (Figure 2D), thus confirming a direct regulation of CD69 expression by HSF1. Nevertheless, the regulation and function of HSF1 upon T cell activation warrants further investigation.

The heat shock response plays critical role in regulating gene expression in response to cellular stress, including inflammation. HSF1 has previously been shown to be involved in the repression of pro-inflammatory cytokines such as IL-1β, TNFα, IFN-γ, as well as activation of the anti-inflammatory gene IL-10 in human monocytes 27, 47. This study aimed to clarify the regulation of CD69 by HSF1. We confirmed the importance of CD69 in the Treg function and we found that HSF1 is critical for CD69 expression and the immunosuppressive function of Tregs. In addition, we reported that CD69^-^ Treg displayed much weaker immunosuppressive function even in the presence of HSF1 expression. Consistent with this, Tregs from *CD69* CKO mice (*HSF1*^+/+^) produced much less IL-10 and TGF-β1 and had weaker immunosuppressive function while HSF1 was still functional (Figure S1E and H). HSF1 inhibition significantly reduced CD69^+^ Tregs differentiation *in vitro* and *in vivo.* As a result, deficiency of HSF1 aggravated colitis in mice, which was reversed after adoptive transfer of iTreg from wild-type mice but not the mice with HSF1 dysfunction. On the other hand, HSF1 has been found to play a critical role in the pathogenesis of various diseases, including cancers, so that inhibitors of HSF1 have been proposed as therapeutics for these disorders 48, 49. Therefore, the potential effect of HSF1 on other inflammation-related genes should not be overlooked. It should take into consideration that inhibition of HSF1 may cause some side effects resulting from the inhibition of CD69^+^ Treg differentiation.

Interestingly, PSIs such as MG132 or bortezomib did not effectively alleviate colitis in mice, indicating that inhibition of proteasomal degradation may have a complicated consequence in many cells other than Tregs *in vivo*. We found that PSIs effectively increased CD69^+^ populations by increasing HSF1 protein in Tregs. The Low stability of iTregs is a major concern for their use in clinical applications. Interestingly, iTregs recovered in the presence of PSIs have a greater capacity to treat colitis in mice. Therefore, the use of PSIs to enhance the induction efficiency of iTregs represents a practical approach to the clinical application of iTregs. We would like to find specific HSF1 stabilizers in the future. Presumably, CD69 is not the only gene affected to increase the potency of iTregs although CD69 knockout greatly attenuated Treg potency, even in the presence of intact HSF1. The contribution of other genes affected by the inhibition of proteasomal degradation to enhanced Treg function warrants further investigation.

Together, HSF1 activates CD69 transcription to promote CD69^+^ Tregs differentiation, which is a Treg subset with potent immunosuppressive function. HSF1 stabilization by PSIs efficiently induces more Tregs with higher potency to treat colitis and probably other autoimmune diseases involving Tregs deficiency.

## Supplementary Material

Supplementary materials and methods, figures and table.

## Figures and Tables

**Table 1:** The sequences of primers and siRNAs

qRT-PCR primers		
Gene	** Forward primer (5'-3')**	**Reverse primer (5'-3')**
CD69	CCCTTGGGCTGTGTTAATAGTG	AACTTCTCGTACAAGCCTGGG
Actin	AACAGTCCGCCTAGAAGCAC	CGTTGACATCCGTAAAGACC
**ChIP qPCR primers**		
**Gene**	**Forward primer (5'-3')**	**Reverse primer (5'-3')**
HSE2	GGATTTTAAGGAACCTTT	CAAGACCGAGGTGCAGGC
**siRNA primers**		
**Gene**	**Forward primer (5'-3')**	**Reverse primer (5'-3')**
1#-siRNA	CUCCUUGAGAAACAUCAAGATT	UCUUGAUGUUCUCAAGGAGTT
2#-siRNA	GACCCAUAAUCUCCGAUAUTT	AUAUCGGGAGAUUAUGGUUCTT
