# Supplementary material for: HSF1 promotes CD69+ Treg differentiation to inhibit colitis progression
Source: Theranostics. 2023 Mar 21;13(6):1892–905. doi: 10.7150/thno.78078 (PMC10091886; doi:10.7150/thno.78078)
Supplement: Supplementary file 1 — Supplementary materials and methods, figures and table. [file thnov13p1892s1.pdf]

Supplementary Materials and Methods

Mice and Cell lines

The female C57BL/6 mice at 6-8 weeks of age were purchased from Joint Ventures Sipper BK Experimental Animal (Shanghai, China). *CD4*^{Cre}and *Foxp3*$^{YFP\text{-}Cre}$mice as described previously were purchased from Cyagen Biosciences. *Rag1*$^{-/-}$ mice were purchased from Model Animal Research Center. All strains of mice were housed in a specific pathogen-free facility. The experimental protocols were approved by the Animal Care and Use Committee of Medical School of Zhejiang University (Hangzhou, China). The CD4^{+}Foxp3/CD25^{+}CD69^{+} and CD4^{+}Foxp3/CD25^{+}CD69^{-} Tregs were sorted using the FACSDiVa system (Becton Dickinson), respectively.

Tregs suppression assay

The suppression of CD4^{+} T cell proliferation in each group was determined by the CFSE incorporation assay. The murine splenic CD4^{+} T cells were isolated using a CD4^{+} T-cell isolation kit II (Miltenyi Biotec, Germany) and were labeled with CFSE (Invitrogen) according to the manufacturer's instruction. CFSE labeled CD4^{+} T cells (1×10^6/ml) were stimulated with 1 μL of anti-CD3/CD28-coated beads (Invitrogen). Purified CD69$^{fl/fl}$-iTregs, CD4^{Cre}CD69$^{fl/fl}$-iTregs at a ratio of 1: 1, 1: 2, or 1:4 were added, the effector CD4^{+} T cells served as the control. Three days later, the cells were harvested, and the proliferation of effector CD4^{+} T cells was analyzed using flow cytometry.

ELISA

IL-10 and TGF-β1 levels in the supernatant of cultured cells and the IL-6, IFN-γ, and IL-17 levels in the colon of mice were measured using specific kits (eBioscience, USA) for ELISA. For analysis of colon explant cultures, the colons of mice were flushed with PBS containing 30% antibiotics, and open along a longitudinal axis. Then, pieces of tissue (~ 3-mm^2) were obtained from the distal colon and incubated for 24 hours in RPMI supplemented with 10% FCS and 20% antibiotics (1 punch biopsy/100 ml medium). Supernatants were collected and kept frozen until assessment. For the detection of TGF-β1**,** 100 μL of supernatants were acidified with 20 μL of 1N HCl at room temperature for 10 min and then neutralized with 20 μL of 1N NaOH to activate latent TGF-β1 into its immunoreactive form.

RNA isolation, cDNA synthesis, and quantitative real-time PCR

Total RNA from the indicated types of cells was extracted using Trizol reagents, and the RNA samples were reverse transcribed into cDNA using a high-capacity cDNA Reverse Transcription Kit (Thermo Fisher, USA). The relative levels of target mRNAs were determined by qRT-PCR. The sequences of primers are listed in Table S1. Data were analyzed using the comparative Ct method using β-actin as the normalization control.

Confocal microscopy

$CD4^+$ cells seeded on chamber slides were cultured overnight. After treatment with MG132 for 2h, cells were fixed with 4% paraformaldehyde and permeabilized in 0.1% Triton X-100/PBS for 10 min. After blocking with 5% bovine serum albumin, cells were incubated with rat anti-HSF1 monoclonal antibody followed by decoration with fluorescein-conjugated anti-rabbit IgG. The images were acquired on confocal microscope.

FACS

To analyze the differentiation of Th1 or Th17 cells, $CD4^+$ T cells were incubated with the cell stimulation cocktail (eBioscience, USA) for 5 h at 37 °C and stained with PE-Cy7-anti-CD4 antibodies. Cells were fixed and stained with PE-anti-IFN-γ (Invitrogen, Clone:XMG1.2; Catalog: 12-7311-82), APC-anti-IL-17 (Invitrogen, Clone: eBio17B7; Catalog: 17-7177-81) antibodies, or isotype controls after permeabilization. The percentages of $CD4^+$IFN-γ^+ and $CD4^+$IL-17^+ cells were determined by flow cytometry.

Protein half-life assay

$CD4^+$ T cells were pretreated with or without MG132 (Sigma Aldrich) for 2 hours then incubated with 50 µg/ml CHX (Sigma Aldrich) for 0,6, 12, 18 hours or 25 before Western blot analysis.

Colitis induction

CD69$^{fl/fl}$ or *Foxp3*$^{YFP\text{-}Cre}$*CD69*$^{fl/fl}$ Mice were orally given 2% DSS (MW 36,000-50,000, MP Biomedicals,USA) in drinking water for 5 days, followed by regular drinking water without DSS. Body weight was measured every 24 h during the

experiment and mice were sacrificed at the indicated day. For T-cell transfer-induced colitis, $CD4^+CD45RB^{hi}$ T-cells from C57BL/6 mice were enriched ($CD4^+$ T-Cell Isolation Kit; Miltenyi Biotec) and single-cell suspensions were stained with APC-anti-CD4 (GK1.5), and PE-anti-CD45RB (C363.16A), all from eBioscience, followed by cell sorting (FACSAriaII) (purification > 99%). $Rag1^{-/-}$ recipient mice received 5 × 10^5 $CD4^+CD45RB^{hi}$ T-cells by intravenous (i.v.) injection, 1 × 10^6 Control-iTregs or PSI-iTregs were injected i.v. 21 days later. At the end of the experiment, the large intestines of individual mice were dissected out and fixed in 10% phosphate-buffered formalin.

Statistical analyses

Data are expressed as the mean ± SD. The difference among the groups was were analyzed by one-way ANOVA and Student *t* test where applicable using GraphPad Prism 8. $P < 0.05$ was considered statistically significant.

Table S1 The sequences of primers

**Real-Time PCR primers**		
**Gene**	**Forward primer（5'-3'）**	**Reverse primer (5'-3')**
IL-10	CCA AGC CTT GGA AA GA	TTT TCA CAG GGG AGA AAT CG
TGF-β1	AACTGCACCCACTTCCCAGTC	CATTAAGGAGTCGGTTAGCAG
HSPA1A	TGGTGCAGTCCGACATGAAG	GCTGAGAGTCGTTGAAGTAGGC
DNAJB1	TTCGACCGCTATGGAGAGGAA	CACCGAAGAACTCAGCAAACA
HSP90AB1	GTCCGCCGTGTGTTCATCAT	GCACTTCTTGACGATGTTCTTGC
Actin	AACAGTCCGCCTAGAAGCAC	CGTTGACATCCGTAAAGACC

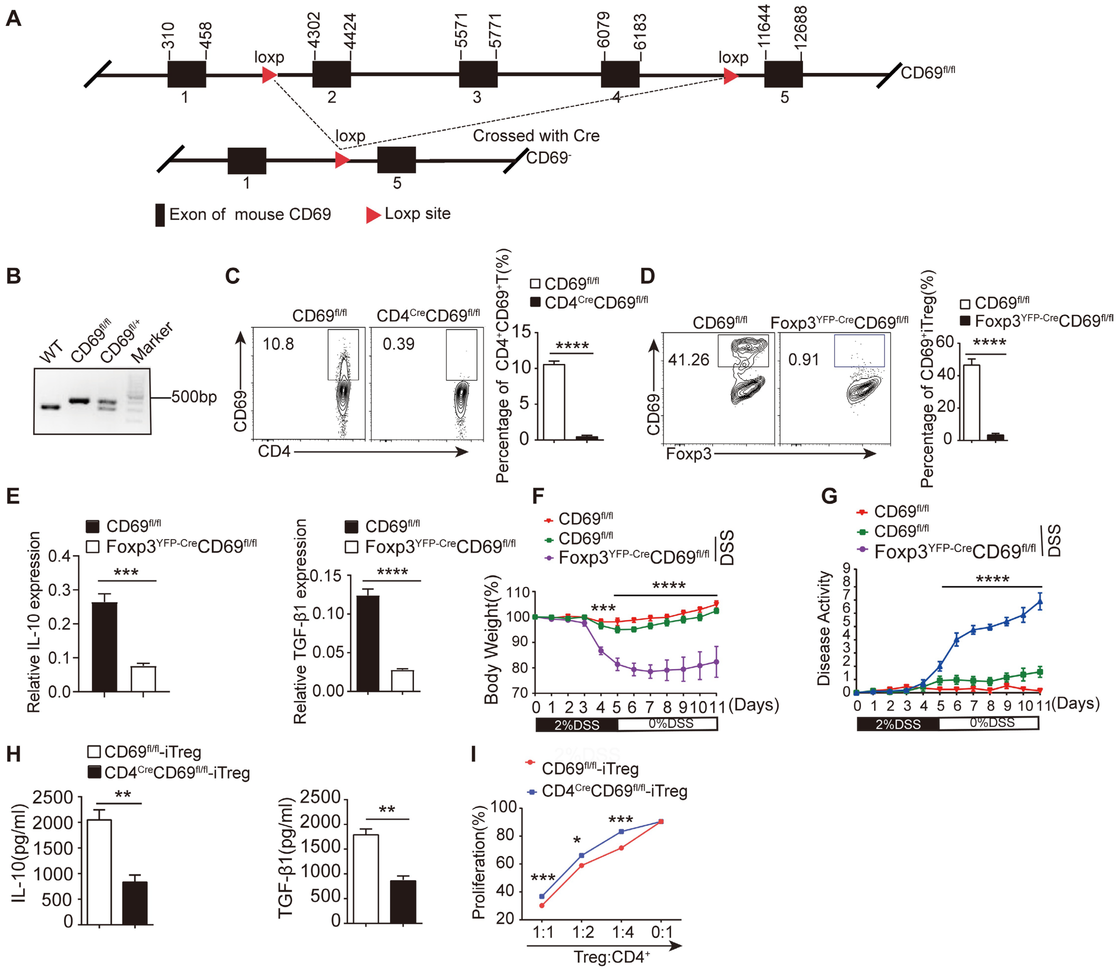

Figure S1. Conditional targeting of the mouse *CD69* gene in Treg exacerbated DSS-induced colitis. (**A**) Strategy of *CD69* conditional knockout mice. (**B**), Genomic DNA was isolated from tail of indicated mice and then used for PCR analysis of *CD69* deletion and the *LoxP*-flanked allele. (**C** and **D**) Phenotype of *CD69*$^{fl/fl}$ × *CD4*^{Cre} *or Foxp3*$^{YFP\text{-}Cre}$ offspring, MLN from conditional knock-out mice were used for analysis the expression of CD69^{+} Tregs. (**E**) The expression of IL-10 and TGF-β1 in Treg from *CD69*$^{fl/fl}$ or *Foxp3*$^{YFP\text{-}Cre}$*CD69*$^{fl/fl}$ mice were analysis by qRT-PCR. (**F** and **G**) *CD69*$^{fl/fl}$ and *Foxp3*$^{YFP\text{-}Cre}$*CD69*$^{fl/fl}$ mice were administrated with 2.0% DSS in drinking water for 5 days, and then they were given by normal water in the following days. Average body weight is shown as percentage relative to initial value. Results are means ± SD (n = 7). (**H**) The IL-10 and TGF-β1 in supernatant of induced Foxp3^{+}CD4^{+}Tregs from *CD69*$^{fl/fl}$ and *CD4*^{Cre}*CD69*$^{fl/fl}$ mice were analysis by ELISA. (**I**) The proliferation of CD4^{+} T cells was analyzed using flow cytometry. The cells

were first gated on living lymphocytes and then on $CFSE^+$ T cells (n = 3). Data are representative of three independent experiments. Comparisons were made using one-way ANOVA or Student's unpaired *t* test. **P* < 0.05; ***P* < 0.01; ****P* < 0.001; *****P* < 0.0001.

A

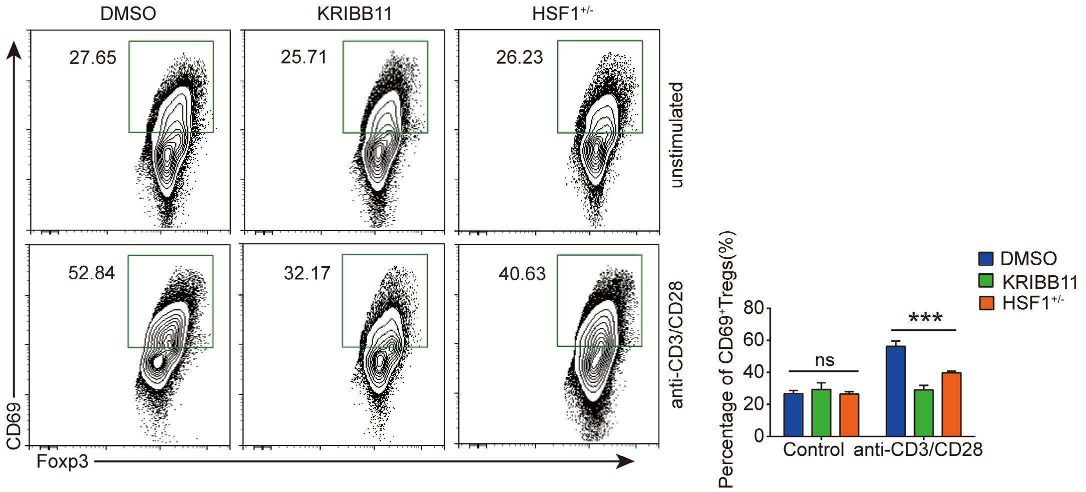

Figure S2. Inhibition of HSF1 reduce CD69 expression of Tregs stimulation with anti-CD3/CD28. (A) 1 × 10^6/ml Tregs were stimulated with or without anti-CD3/CD28 antibodies and treated with KRIBB11for the indicated time. Relative levels of CD69 expression in different groups of Tregs were determined by FACS. Results are means ± SD. *** *p* < 0.001, as analyzed by Student's unpaired *t* test.

A

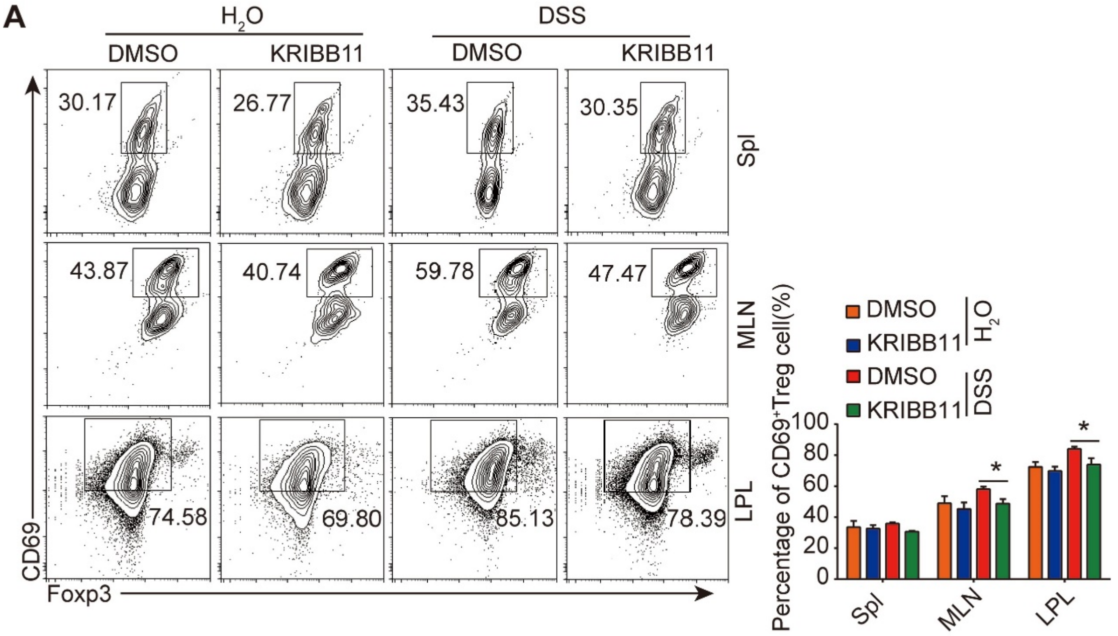

Figure S3. The differentiation of CD69$^+$ Treg cells is inhibited in DSS induced colitis mice treated with KRIBB11. (**A**) Spleen, MLN and colonic lamina propria cells were isolated from control or mice injected with HSF1 inhibitor KRIBB11 after administration of DSS for 5 days. The frequency of Tregs and CD69$^+$ Tregs was detected by flow cytometry. The graph shows the average percentage of Foxp3$^+$cells among CD4$^+$ T cells and CD69$^+$ cells among Tregs. Results are means ± SD (n = 5 mice per each group). *$p < 0.05$, as analyzed by Student's unpaired *t* test.

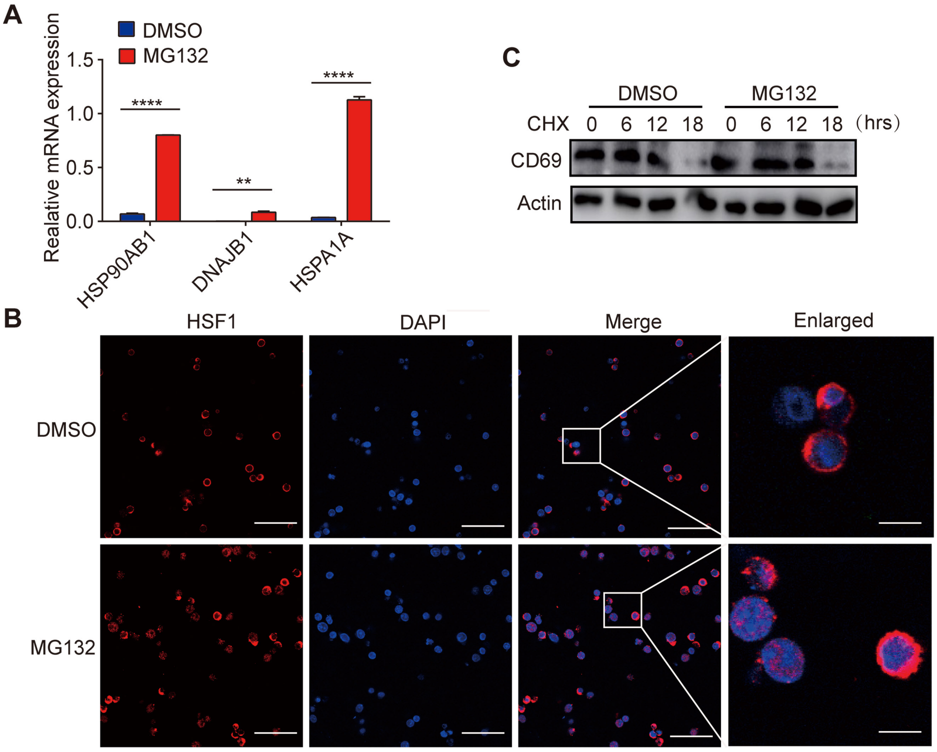

Figure S4. MG132 stabilized HSF1 protein to activate the transcription of its target genes. (**A**) *HSP90AB1*, *HSP90A1A* and *DNAJB1* normalized to β-actin were analyzed by qRT-PCR; (**B**) CD4$^+$ T cells were treated with MG132 for 2 h, HSF1 immunoflurescence staining was performed. (**C**) CD4$^+$ T cells were treated with CHX alone or pretreatment with MG132 for time periods as indicated and CD69 protein level was measured by western blotting. The date are presented as the means ± SD of three repeated experiments. **$p < 0.01$, ****$p < 0.0001$ as analyzed by Student's unpaired *t* test.

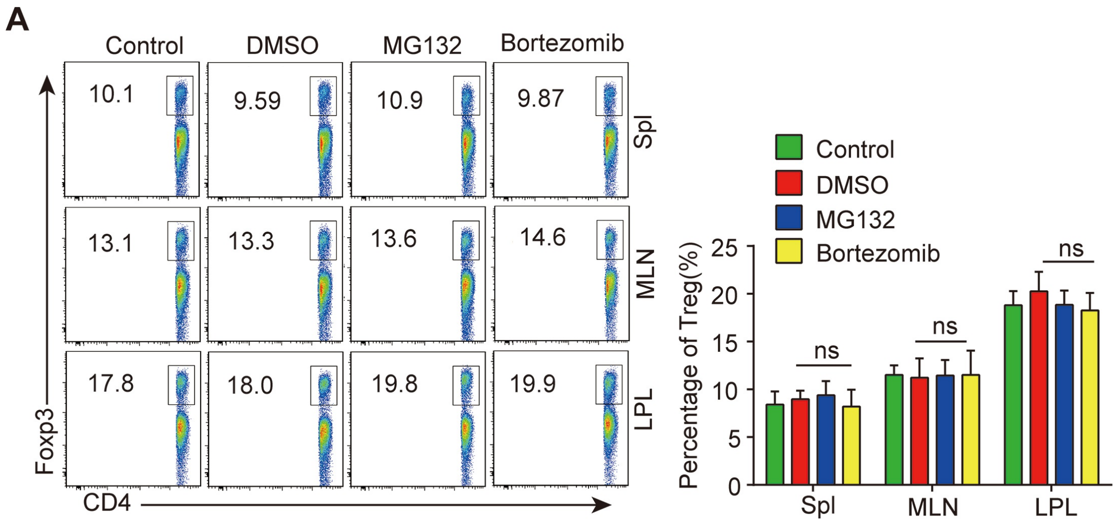

Figure S5. (A) Density plots showing total CD4$^+$Foxp3$^+$ cells from freshly isolated spleen, MLN and colonic LPL in mice treated with MG132 and Bortezomib. Representative images of the data expressed as mean ± SD of three independent experiments. ns, not significant, as analyzed by ANOVA or Student's *t*-test.

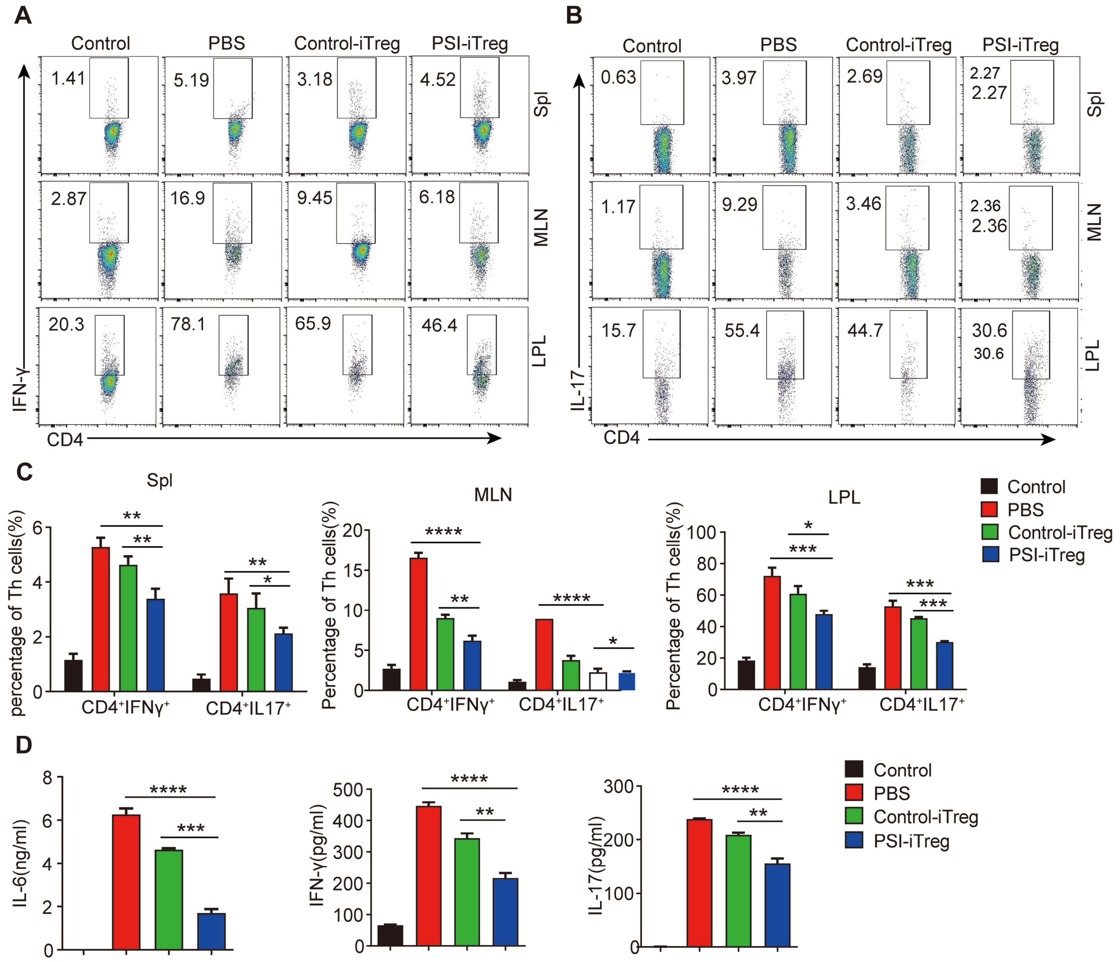

Figure S6. PSI-iTreg prevents mice from exacerbation of DSS-induced colitis. (**A-C**) Each group of T cells was stimulated with the cell stimulation cocktail for 6 h and then stained with anti-mouse CD4 and anti-IFN-γ or anti-IL-17 antibodies, followed by flow cytometry. (**D**) Colon tissue was cultured overnight, and cytokines in the supernatant were measured by ELISA (n = 5). Representative images of the data expressed as the mean ± SD of three independent experiments. **p* < 0.05, ***p* < 0.01, ****p* < 0.001, *****p* < 0.0001, as analyzed by One-way ANOVA or Student's unpaired *t* test.

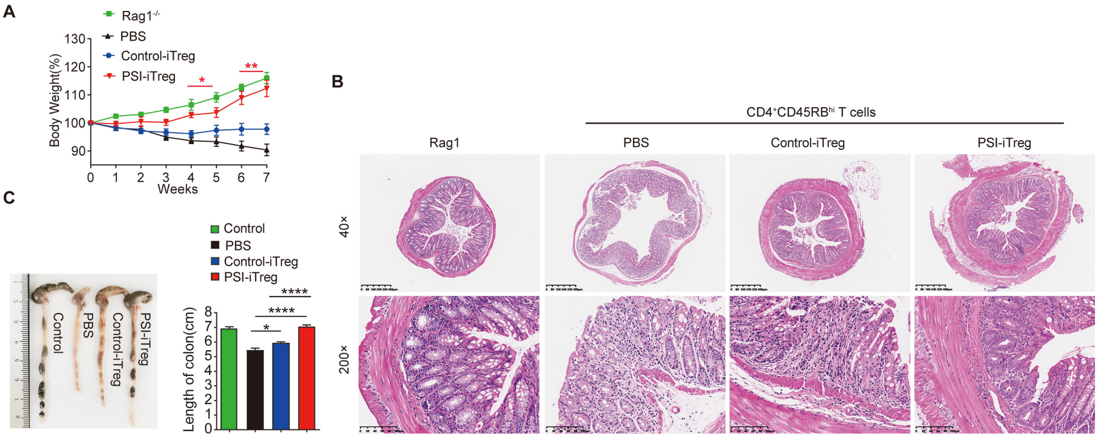

Figure S7. Adoptive transfer of PSI-iTregs inhibits the disease progression in a mouse model of T-cell transfer-induced colitis. Spleen cells from C57BL/6 mice were enriched for $CD4^+$ T-cells and then stained with anti-CD4 and anti-CD45RB monoclonal antibodies. Then $CD4^+Foxp3^-CD45RB^{hi}$ T-cells were sorted and injected i.v into immunodeficient *Rag1*$^{-/-}$ mice. Groups of mice were injected i.v with Control-iTregs and PSI-iTregs (1 × 10^6/mouse/injection) on days 21 (n = 7). (**A**) The body weights were measured for 7 weeks. Each point represents average weight data pooled from 7 mice ± SD. (**B**) Histological appearance 7 weeks after colitis induction. **(C)** Appearance and statistical analysis of colon length. Representative colonic sections stained with H&E (Magnification: 40 × and 200 ×). Date are representative images or expressed as the mean ± SD of three independent experiments (n = 7 per group). **P* < 0.05, ***P* < 0.01, analyzed by ANOVA and Student's t-test.
